# Effects of Dietary Energy Levels on Rumen Fermentation, Microbial Diversity, and Feed Efficiency of Yaks (*Bos grunniens*)

**DOI:** 10.3389/fmicb.2020.00625

**Published:** 2020-05-15

**Authors:** Anum Ali Ahmad, Chao Yang, Jianbo Zhang, Qudratullah Kalwar, Zeyi Liang, Chen Li, Mei Du, Ping Yan, Ruijun Long, Jianlin Han, Xuezhi Ding

**Affiliations:** ^1^Key Laboratory of Veterinary Pharmaceutical Development, Ministry of Agricultural and Rural Affairs and Key Laboratory of Yak Breeding Engineering, Lanzhou Institute of Husbandry and Pharmaceutical Sciences, Chinese Academy of Agricultural Sciences, Lanzhou, China; ^2^State Key Laboratory of Grassland Agro-Ecosystems, School of Life Sciences, Lanzhou University, Lanzhou, China; ^3^CAS Key Laboratory for Agro-Ecological Processes in Subtropical Region, National Engineering Laboratory for Pollution Control and Waste Utilization in Livestock and Poultry Production, Hunan Provincial Engineering Research Center for Healthy Livestock and Poultry Production, South-Central Experimental Station of Animal Nutrition and Feed Science in Ministry of Agriculture, Institute of Subtropical Agriculture, Chinese Academy of Sciences, Changsha, Hunan, China; ^4^CAAS-ILRI Joint Laboratory on Livestock and Forage Genetic Resources, Institute of Animal Science, Chinese Academy of Agricultural Sciences, Beijing, China; ^5^Livestock Genetics Program, International Livestock Research Institute, Nairobi, Kenya

**Keywords:** yak, dietary energy, rumen microbiota, volatile fatty acid, feed efficiency, growth performance

## Abstract

The microbial community of the yak (*Bos grunniens*) rumen plays an important role in surviving the harsh Tibetan environment where seasonal dynamic changes in pasture cause nutrient supply imbalances, resulting in weight loss in yaks during the cold season. A better understanding of rumen microbiota under different feeding regimes is critical for exploiting the microbiota to enhance feed efficiency and growth performance. This study explored the impact of different dietary energy levels on feed efficiency, rumen fermentation, bacterial community, and abundance of volatile fatty acid (VFA) transporter transcripts in the rumen epithelium of yaks. Fifteen healthy castrated male yaks were divided into three groups and fed with low (YL), medium (YM), and high energy (YH) levels diet having different NEg of 5.5, 6.2, and 6.9 MJ/kg, respectively. The increase in feed efficiency was recorded with an increase in dietary energy levels. The increase in dietary energy levels decreased the pH and increased the concentrations of acetate, propionate, butyrate, and valerate in yak rumens. The increase in the mRNA abundance of VFA transporter genes (*MCT1*, *DRA*, *PAT1*, and *AE2*) in the rumen epithelium of yaks was recorded as dietary energy level increased. High relative abundances of Firmicutes and Bacteroidetes were recorded with the increase in dietary energy levels. Significant population shifts at the genus level were recorded among the three treatments. This study provides new insights into the dietary energy-derived variations in rumen microbial community.

## Introduction

During recent years, the manipulation of rumen microbiota by altering diet to improve animal growth performance has gained increasing attention. The rumen is a complex environment containing diverse microbes that assist the host in digesting and utilizing feed energy. Rumen microbes produce various glucanases and xylanases to digest solid fiber by adhering to its surface, finally converting it into volatile fatty acids (VFAs), that is, acetate, propionate, and butyrate (Luo et al., 2001; Kamra, 2005). VFAs are the main source of energy in ruminants, as they provide 70–80% of the body’s energy needs and aid in growth and production performance. The VFAs produced in the rumen are absorbed and transported through the rumen epithelium into the blood by VFA transporters (Van Houtert, 1993; Yohe et al., 2019). So far, VFA transporters, including monocarboxylic acid transporters (MCT1), anion exchange carriers (AE2), downregulated in adenoma (DRA) proteins, and putative anion transporters (PAT1) are reported to be actively involved in VFA transportation through the rumen epithelium (Rajendran et al., 2000; Foltz et al., 2004).

Yak rumen microbiome coevolved with the host genome to adapt to extreme environmental conditions in the Qinghai Tibetan Plateau, where dynamic seasonal changes in the pasture cause nutrient supply imbalances, resulting in weight loss in yaks (Xue et al., 2005). Yaks greatly depend on metabolites produced by microbes in the rumen for their growth and survival (Saleem et al., 2013). Therefore, a better understanding of rumen microbiota under different feeding regimes is important for exploiting the microbiota to enhance feed efficiency (Jami and Mizrahi, 2012; Bannink et al., 2016). Feed efficiency is an important management tool in yak husbandry to improve economics of meat and milk production (Xu et al., 2017).

An increase in dietary energy levels displayed promising results in the performance of 3-year-old male yaks. Yaks showed average daily weight gains of 286.91, 446.75, and 770.42 g/day when fed with diets of 3.72, 4.52, and 5.32 MJ/kg, respectively (Hongze, 2015). A study conducted on yaks reported the difference in rumen microbiota and certain metabolites when fed different feed types divided into forage and concentrate groups (Liu et al., 2019). However, little information is available regarding the impact of different dietary energy levels on dynamic changes in the rumen fermentation and microbiota and their influence on transporter genes in the rumen epithelium of yaks. The objectives of this study were to evaluate the effect of three dietary energy levels on feed efficiency, rumen fermentation, and bacterial composition of yak. Moreover, we also studied the effects of different dietary energy levels on transcript abundance of VFA transporter genes in the rumen epithelium of yaks.

## Materials and Methods

### Ethics Statement

All procedures involving animal care and use were in strict accordance with the *Guide for the Care and Use of Laboratory Animals*, Lanzhou Institute of Husbandry and Pharmaceutical Sciences, CAAS, China [SCXK (Gan) 2014-0002]. After experiment completion, all yaks were transferred to a facility where they were humanely slaughtered by electrical stunning and exsanguination.

### Animals, Diets, and Experimental Design

The feeding trial was conducted from February to May 2016 at Hongtu Yak Breeding Cooperatives of Tibetan Autonomous Prefecture in Gannan, Gansu Province, China. In brief, 15 adult castrated male yaks with similar body conditions were randomly divided into three treatment groups. Three diets of different net energy levels and a concentrate-to-forage ratio of 30:70 (DM basis) were formulated, containing similar roughage mixtures (40% microbial corn stalk silage, 40% oat silage, and 20% highland barley hay) and different energy concentrates: low (YL: 5.5 MJ/kg), medium (YM: 6.2 MJ/kg), and high energy levels (YH: 6.9 MJ/kg). The ingredients and nutrient compositions of the three diets are shown in Table 1 (Yang et al., 2017). The experiment lasted for 60 days after a 14-day adaptation period. The animals were fed *ad libitum* twice daily at 08:00–09:00 and 17:00–18:00 with a total of 2.45-kg concentrate and 5.75-kg roughage mixtures with free access to water. The orts were recorded to calculate average daily feed intake (ADFI), and ADFI was determined as the total feed intake/60 (Yang et al., 2017, 2019). Animals of each group were weighed before the morning feeding on the first and 60th days of the trial; feed efficiency was calculated as average daily gain/ADFI during the experimental period.

**TABLE 1:** Ingredients and nutrient composition of three concentrates used during the experiment.

Item	Treatment^1^		
	YL	YM	YH
**Ingredient, g/kg of DM**			
Corn	290	448	560
Corn germ	300	200	120
Wheat bran	40	40	–
DDGS^2^	150	70	63
Prickly ash seed	100	20	40
Cottonseed meal	60	120	160
Soybean meal	–	50	–
Salt	8	8	8
White stone powder	20	20	20
Dicalcium phosphate	6	6	6
Urea	8	–	5
Sodium bicarbonate	10	10	10
Premix^3^	8	8	8
**Nutrient composition, g/kg of DM**			
Crude protein	165.3	167.4	172.1
Crude fat	37.3	41.8	55.7
NEg^4^ (MJ/kg DM)	55	62	69
Neutral detergent fiber	159.3	131.5	123.2
Acid detergent fiber	45.4	41.4	37.2
Calcium	6.4	8.4	7.5
Phosphorus	3.1	3.4	3.6

*^1^LE, low energy level; ME, medium energy level; HE, high energy level. ^2^DDGS, distillers dried grains with solubles. ^3^Premix was provided per kilogram of total diet DM, and the composition was as follows: 22,520 IU of vitamin A, 1920 IU of vitamin D_3_, 18 IU of vitamin E, 0.36 IU of vitamin K_3_, 5.28 mg of vitamin B_2_, 0.008 mg of vitamin B_12_, 21.2 mg of D-calcium pantothenate, 9 mg of Cu, 132.8 mg of Zn, 240 mg of Fe, 8 mg of Mn, and 0.28 mg of Co. ^4^NEg, net energy for gain, is calculated according to Feeding Standard of Beef Cattle (NY/T 815-2004); others were measured values.*

### Sample Collection

At the end of the experiment, rumen fluid (20 ml) from yaks of each group was extracted via an oral stomach tube before the morning feeding on day 60. The tube was thoroughly cleaned using fresh warm water between sample collections, and 10–15 ml of the sample from each yak was always discarded to avoid contamination from saliva. The rumen fluid was immediately frozen in liquid nitrogen and then stored at −80°C for later analysis of VFA and bacterial diversity. Yaks were then transferred to a facility and humanely slaughtered by electrical stunning and exsanguination. The rumen was immediately separated and placed on dry ice; the rumen epithelial tissues of ∼10-cm^2^ size were excised from the caudoventral sac and washed with ice-cold phosphate buffer saline (pH = 7.0) to remove plant particles. The samples were snap frozen in liquid nitrogen and later stored at −80°C for total RNA isolation.

### Determination of Rumen Fermentation Parameters

The rumen fluid pH was measured using a portable pH meter (Sartorius PB-10, Sartorius, Göttingen, Germany) during fluid collection. The cryopreserved rumen fluid sample was thawed at 4°C and thoroughly mixed by vortexing. Next, 10 ml of the rumen fluid was taken and centrifuged at 3000 × *g* for 10 min; then, 1 ml of supernatant was placed in a 1.5-ml centrifuge tube, and 0.2 ml of a metaphosphoric acid solution containing the internal standard 2-ethylbutyric acid was added. The sample was mixed, placed in an ice water bath for 30 min, and centrifuged at 10,000 × *g* at 4°C. The supernatant was placed in a new 1.5-ml centrifuge tube and stored in a 4°C refrigerator for testing. The VFA concentration was determined by gas chromatography (Varian 450, Agilent Technologies China, Co., Ltd., China). The gas chromatographic conditions and subsequent test procedures were conducted as described previously (Horwitz, 2010).

### Rumen Bacterial Diversity Analysis

Genomic DNA was extracted from rumen fluid samples by the cetyltrimethylammonium bromide method (William et al., 2012). The DNA concentration was determined by NanoDrop 2000 (Thermo Fisher Scientific, Waltham, MA, United States), and its integrity was checked by 1.2% agarose gel electrophoresis. The extracted DNA was used for PCR amplification of the V4 region of the 16S rRNA gene using universal primer pairs (515F–806R) with barcodes. PCR amplification was performed using the Phusion^®^ High-Fidelity PCR Master Mix with GC Buffer (New England BioLabs, Ipswich, MA, United States). The amplified products were subjected to 1.5% agarose gel electrophoresis and gel purified by using the QIAquick PCR Purification Kit to construct libraries. The libraries were constructed using the TruSeq^®^ DNA PCR-Free Sample Preparation Kit (Illumina, San Diego, CA, United States) according to the manufacturer’s protocol. The constructed libraries were quantified by the Qubit dsDNA High-Sensitivity Assay Kit (Invitrogen, Carlsbad, CA, United States), and paired-end sequencing was performed using the Illumina HiSeq 2500 PE250 system (Illumina, San Diego, CA, United States) according to standard protocol. The sequencing data were analyzed by using the QIIME (Quantitative Insights Into Microbial Ecology, Version 1.7.0) pipeline. All sequence reads were trimmed and assigned to each sample based on their barcodes. High-quality sequences were clustered into operational taxonomic units (OTUs) at 97% identity using UCLUST software Version 7.1^1^. The QIIME software was used to calculate Chao1, Shannon, Simpson, ACE, and Good’s coverage indices, while R software (Version 2.15.3) was used to construct weighted UniFrac distance-based principal coordinates analysis (PCoA) plot to illustrate significance differences between samples.

### Analysis of the Transcripts Abundance of VFA Transporter Genes

Total RNA was isolated from the rumen epithelium by the TaKaRa MiniBEST Universal RNA Extraction Kit (code no. 9767; TaKaRa, Dalian, China) according to the manufacturer’s instructions. The quality and integrity of total RNA were determined by NanoDrop 2000 (Thermo Fisher Scientific, Waltham, MA, United States) and 1.2% agarose gel electrophoresis, respectively. The RNA samples were reverse transcribed using the PrimeScript^TM^ RT Reagent Kit (TaKaRa, Dalian, China) according to the manufacturer’s instructions. Primers were designed using the Primer Premier 5 software (PREMIER Biosoft International, San Francisco, CA, United States) for VFA transporter genes *MCT1*, *DRA*, *PAT1*, and *AE2* (Supplementary Table S1). Quantitative real-time PCR (qRT-PCR) was performed in triplicate to determine the transcripts’ relative abundances using SYBR1 Premix Ex Taq^TM^ II (TaKaRa, Dalian, China) relative to transcript levels of reference gene β-actin. Reactions were run on a fluorescence thermal cycler (CFX96, Bio-Rad, Hercules, CA, United States), and the program was as follows: 95°C for 30 s, 40 cycles of 95°C for 5 s and annealing at 60°C for 30 s, and a melting curve with a temperature increase of 0.5°C every 5 s starting at 65°C. qRT-PCR analysis for each studied gene was performed using cDNA from five biological replicates with three technical replicates per biological replicate. The threshold cycle (CT) resulting from qRT-PCR was analyzed using the 2^–Δ^ ^Δ^ ^*Ct*^ method, and all data were normalized with the reference gene β-actin.

### Statistical Analysis

The R software (Version 2.15.3) was used to perform variation tests among three treatments at various classification levels (phylum and genus) to obtain *P*-values. The lm function of the Estimability package in R was used to evaluate the linear and quadratic effects of the dietary energy levels on the bacterial abundance. Analysis of similarities (Anosim) was executed using the Anosim function of the R vegan package. The growth parameters, VFA concentration data, and abundance of transcripts of VFA transporter genes were processed by one-way analysis of variance using the least significant difference procedure to perform multiple comparisons in the SPSS software. Significance was declared at *P* < 0.05. Pearson correlations analysis was performed to calculate relations of VFA concentration and abundance of transcripts of VFA transporter genes with relative abundance of top bacterial genera having a relative abundance of ≥0.1% in at least one of the samples using GraphPad Prism 8.0.2, and a heatmap was generated by R software (Version 2.15.3). *P*-values were adjusted with false discovery rate, and the corrected *P*-values < 0.05 were regarded as statistically significant.

### Data Availability

The data for this study have been deposited in the European Nucleotide Archive (ENA) at EMBL-EBI under accession number PRJEB34298^2^.

## Results

### Yak Growth Performance

The growth performance of yaks has been previously reported (Yang et al., 2019). The final body weights of YM and YH group animals were significantly higher than those in the YL group (*P* < 0.05). However, no significant increase in the final body weight was observed between YM and YH groups (*P* < 0.05). Feed efficiency was significantly affected (*P* < 0.001) by an increased dietary energy level and was higher in the YM and YH groups than in the YL group; however, no significant difference was observed between YH and YM groups (Table 2).

**TABLE 2:** Effects of dietary energy levels on growth performance of yak.

Parameters	Treatment groups			SEM	*P*-value
	YL	YM	YH		
Initial BW (kg)	276.0	277.0	275.2	2.55	0.78
Final BW (kg)	313.4^*b*^	330.0^*a*^	326.8^*a*^	4.35	0.037
Average daily gain (kg/d)	0.63 ^*b*^	0.88^*a*^	0.86^*a*^	0.034	<0.001
Average daily feed intake (ADFI) (kg/d)	8.12^*a*^	7.96	7.78^*b*^	0.05	0.01
Feed efficiency	0.076^*a*^	0.11^*b*^	0.11^*b*^	0.01	<0.001

*YL, low energy; YM, medium energy; YH, high energy; SEM, standard error of the mean. Values are presented as mean ± SEM. Means in a row with different small letter superscripts differ significantly (*P* < 0.05); those with the same letter superscripts present no difference (*P* > 0.05).*

### Rumen VFA Concentrations and Transcript Abundances of VFA Transporter Genes

**FIGURE 1:**
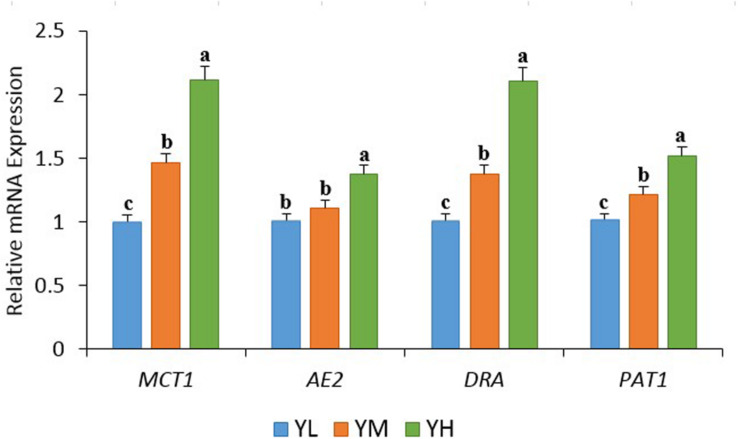
The transcript abundances of VFA transporter genes in the rumen epithelium of yak among three dietary energy treatments. Within a panel, means without a common letter differ (*P* < 0.05). *MCT1*, monocarboxylate transporter 1; *DRA*, downregulated in adenoma; *AE2*, anion exchanger 2; *PAT1*, putative anion transporter 1.

The increase in dietary energy level significantly decreased the pH of yak rumen fluid (*P* < 0.05), and significant increases in the concentrations of acetate, propionate, butyrate, valerate, and total VFA were observed (*P* < 0.05) (Table 3). The influence of different dietary energy levels was investigated by examining the transcript abundance of VFA transporter genes (Figure 1). The increase in dietary energy levels significantly enhanced (*P* < 0.05) the mRNA abundance of *MCT1*, *DRA*, and *PAT1* in the yak rumen epithelium. Abundance of *AE2* transcripts showed no difference between YL and YM groups (*P* > 0.05) but was significantly higher in the YH group (*P* < 0.05).

**TABLE 3:** Effects of different dietary energy levels on rumen VFA concentrations of yak.

Parameters	Treatment groups			SEM	*P*-value
	YL	YM	YH		
pH	6.90^a^	6.66^b^	6.47^c^	0.05	<0.001
Total VFA (mM)	51.4^c^	60.9^b^	72.1^a^	2.41	<0.001
Acetate (mM)	37.4^c^	44.9^b^	51.9^a^	1.71	<0.001
Propionate (mM)	8.7^c^	9.7^b^	11.4^a^	0.36	0.001
Butyrate (mM)	4.02^c^	4.98^b^	7.11^a^	0.45	<0.01
Isobutyrate (mM)	0.32	0.33	0.37	0.01	0.08
Valerate (mM)	0.26^c^	0.31^b^	0.44^a^	0.02	<0.01
Isovalerate (mM)	0.66	0.66	0.77	0.02	0.09

*YL, low energy; YM, medium energy; YH, high energy; SEM, standard error of the mean. Values are presented as mean ± SEM. Means in a row with different small letter superscripts differ significantly (*P* < 0.05); those with the same letter superscripts present no difference (*P* > 0.05).*

### Bacterial 16S rRNA Gene Sequencing

In total, 911,966 raw reads (average 59,192 sequences per sample) were obtained from bacterial 16S rRNA sequencing of 15 samples. After quality filtering, 895,658 sequences were obtained, of which 7783 were chimeric reads that were removed from further analysis. The mean sequence read length was 253 bp. The non-chimeric reads were clustered into 2662 OTUs at 97% similarity levels, of which 2118 were identified as low-abundance OTUs (79.6%) having less than 10 reads, and 749 OTUs were present in all three treatment groups (28%). The qualified sequences were clustered at 97% similarity levels. Good’s coverage for all samples was more than 99%. The sequencing depth to describe the OTU-level bacterial diversity was evaluated by a rarefaction curve of observed species of all samples (Supplementary Figure S1). The curves of all samples reached a plateau, indicating that a sufficient number of sequences had been generated to investigate bacterial diversity in the rumen. OTU analysis identified 1828 common OTUs in three groups, while 314, 67, and 69 unique OTUs were discovered in the YL, YM, and YH groups, respectively.

Of the 26 identified bacterial phyla in the rumen samples, Firmicutes, Bacteroidetes, Tenericutes, and Lentisphaerae showed high relative abundance among the three groups. The mean relative abundances of Firmicutes and Bacteroidetes were 48.4 ± 5.7 and 39.4 ± 5.4%, respectively, accounting for 87.8% of the total phyla (Supplementary Figure S2), and were higher in YM and YL groups, respectively. A high Firmicutes: Bacteroidetes F:B ratio (1.41) was recorded in the YM group, followed by YH (1.27) and YL (1.12); however, these changes were not significant among the three groups (Table 4). Other phyla, that is, Proteobacteria, Chloroflexi, Fibrobacteres, and Spirochaetes had relative abundances of ≥0.1% in all groups. These predominant phyla displayed different relative abundances and compositions among the three groups (Supplementary Figure S2). Different dietary energy levels had little influence on relative abundances of the major phyla; however, Tenericutes (*P* = 0.04), Lentisphaerae (*P* = 0.02), Spirochaetes (*P* = 0.02), and SHA-109 (*P* = 0.02) showed significant differences. Lentisphaerae, Spirochaetes, and SHA-109 were linearly increased as the diet energy level changed from low to high, while Tenericutes showed a quadratic relationship (Table 4).

**TABLE 4:** Main phylum of the rumen samples from different dietary treatment groups of yak.

Phylum	Treatment groups			SEM	*P*-value
	YL	YM	YH		
Firmicutes	0.46	0.51	0.48	0.014	0.52
Bacteroidetes	0.43	0.36	0.38	0.014	0.16
Tenericutes	0.03^ab^	0.05^a^	0.02^b^	0.004	0.04
Lentisphaerae	0.026^b^	0.03^b^	0.04^a^	0.003	0.02
Proteobacteria	0.015	0.012	0.012	0.001	0.72
Chloroflexi	0.004	0.002	0.007	0.001	0.31
Fibrobacteres	0.005	0.006	0.004	0.0008	0.70
Spirochaetes	0.003^b^	0.004^b^	0.008^a^	0.0008	0.02
SHA-109	0.0004^b^	0.0005^b^	0.001^a^	0.0002	0.02
F:B	1.12	1.41	1.27	0.082	0.39

*YL, low energy; YM, medium energy; YH, high energy; SEM, standard error of mean. Means in a row with different small letter superscripts differ significantly (*P* < 0.05).*

**FIGURE 2:**
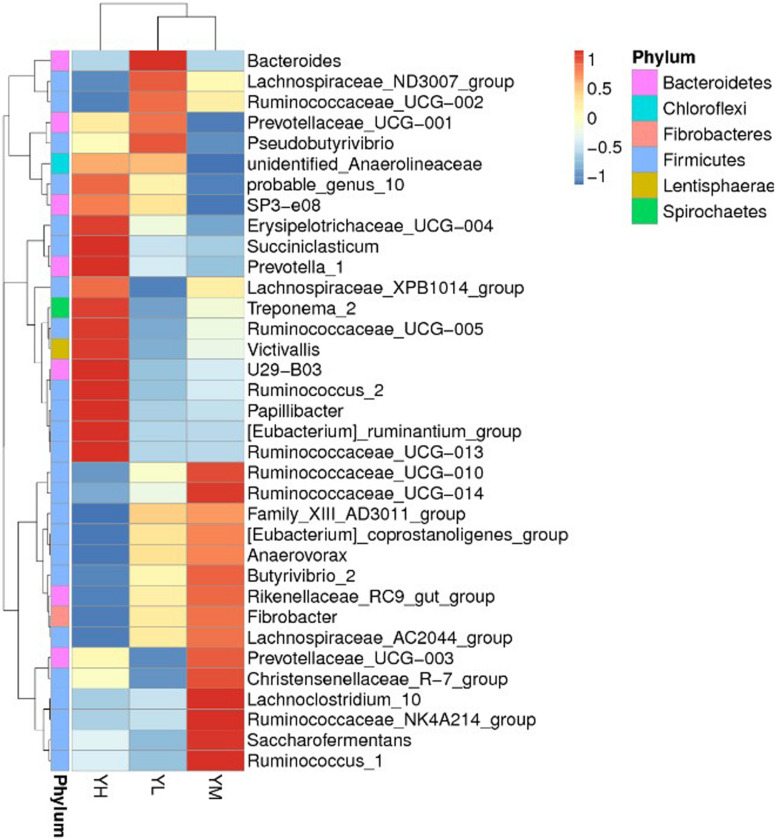
Heatmap showing the rumen bacterial composition at genus level in yak fed different dietary energy levels. YL, low energy; YM, medium energy; YH, high energy.

At the genus level, 274 genera were identified in all samples, of which *Christensenellaceae_R-7_group*, *Rikenellaceae_RC9_gut_group*, *Prevotella_1*, *Ruminococcaceae_NK4A214_group*, and *Prevotellaceae_UCG-003* were the most abundant. The mean relative abundances of *Christensenellaceae_R-7_group* and *Rikenellaceae_RC9_gut_group* were 12.4 ± 4 and 10.9 ± 3.2%, respectively, accounting for 23.3% of the total genera (Figure 2). A heatmap was constructed to examine the relative abundance of different genera in the three treatment groups (Figure 2). The relative abundances of *Bacteroides* and *Pseudobutyrivibrio* were higher in YL than in YM and YH groups. High relative abundances of *Rikenellaceae_RC9_gut_group*, *Ruminococcaceae*, *Ruminococcus*, *Lachnoclostridium*, and *Saccharofermentans* were recorded in the YM group, while *Erysipelotrichaceae*, *U29-B03*, *Succiniclasticum*, *Prevotella*, *Treponema*, *Victivallis*, and *Papillibacter* were the most abundant in the YH group.

### Bacterial Diversity Analysis

Significant differences were found in the bacterial communities at the OTU level between YH and YL (Anosim, *P* < 0.05) groups and YH and YM (*P* < 0.01) groups, while no significant difference was observed between the YM and YL (*P* > 0.05) groups. For alpha diversity analysis, we calculated the Shannon diversity, Simpson, ACE, and Chao1 indices for each treatment. Alpha diversity analysis showed no significant (*P* > 0.05) difference in the rumen bacterial community as dietary energy levels increased (Table 5). The rumen bacterial diversity of each group tended to be stable, but YM group diversity indices were slightly higher than those of the YL and YH groups.

**TABLE 5:** Alpha diversity indices for different dietary treatment groups of yak.

Indices	Treatment groups			SEM	*P*-value
	YL	YM	YH		
Shannon	8.09	8.18	8.17	0.03	0.49
Simpson	0.99	0.99	0.99	0.0006	0.05
Chao1	1721.6	1732.4	1624.3	32.50	0.35
ACE	1720.4	1736.6	1646.5	27.07	0.37

*YL, low energy; YM, medium energy; YH, high energy; SEM, standard error of the mean.*

**FIGURE 3:**
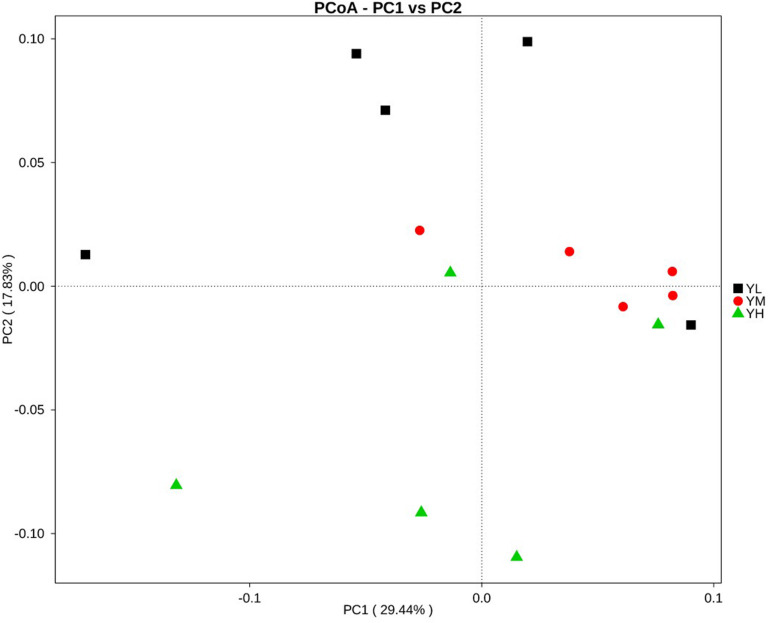
Principal coordinate analysis (PCoA) for three dietary energy treatments. *X*-axis, first principal component, and *Y*-axis, second principal component. Different colors represent different groups.

Principal coordinates analysis for beta diversity showed that the bacterial composition and structure under different energy diets were significantly different, as indicated by the first two principal component scores that accounted for 29.44 and 17.83% of the total variations (Figure 3).

### Correlation of Bacterial Genera With VFA Concentrations and Transcript Abundances of VFA Transporter Genes

**FIGURE 4:**
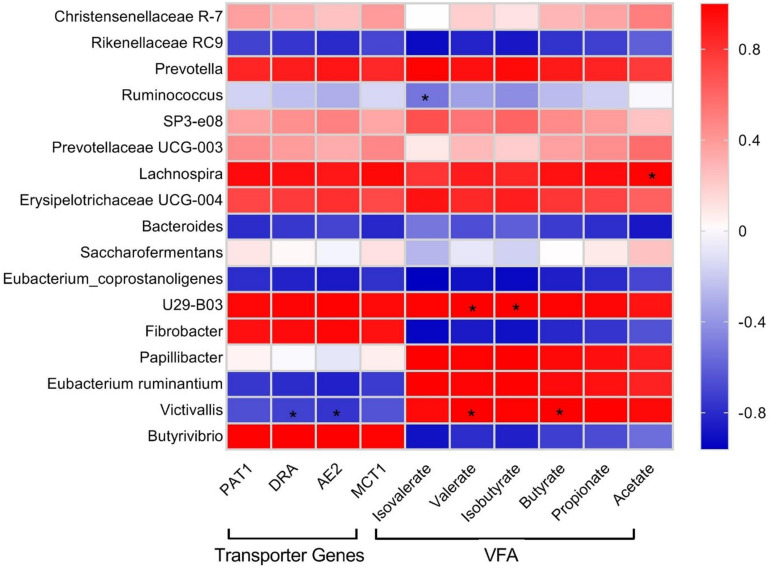
Pearson correlation coefficients for microbial genera with rumen VFA concentrations and transcript abundances of VFA transporter genes in yak. Color intensity represents correlation coefficient. Significant difference (*P* < 0.05) is represented by an asterisk (*).

Pearson correlation analysis was performed in order to check the relationships of the relative abundance of selected bacterial genera identified by 16S rRNA sequencing with rumen VFA concentration and abundance of VFA transporter transcripts (Figure 4). *Erysipelotrichaceae UCG-004*, *Lachnospira*, *Prevotellaceae UCG-003*, *SP3-e08*, *Prevotella*, *U29-B03*, and *Christensenellaceae_R-7* showed positive correlations with VFA and VFA transporter genes. *Papillibacter* displayed a strong positive correlation with VFA, weak positive correlation with *PAT-1* and *MCT-1*, and weak negative correlation with *DRA* and *AE2*. *Eubacterium ruminantium* and *Victivallis* showed strong positive correlations with VFA and strong negative correlations with VFA transporter genes. *Saccharofermentans* showed weak positive correlations with acetate, propionate, butyrate, *PAT-1*, *DRA*, and *MCT-1* and weak negative correlations with isobutyrate, valerate, isovalerate, and *AE2*. *Butyrivibrio* and *Fibrobacter* displayed strong negative correlations with both VFA and VFA transporter genes. *Eubacterium_coprostanoligenes*, *Bacteroides*, *Ruminococcus*, and *Rikenellaceae_RC9* exhibited negative correlations with VFA and VFA transporter genes.

## Discussion

Feed efficiency is an important trait for yak husbandry and economic income. Feed efficiency is influenced by many factors such as management practices, physiological mechanism, nutrition, host genetics, and rumen microbiota. Rumen microbiota provides feed energy to the animal through fermentation in the form of VFA. Studies have shown that rumen microbiota can be manipulated by diet to enhance feed efficiency of animals (Jiao et al., 2015; Yang et al., 2017). So, in this study, we evaluated feed efficiency, rumen bacterial community, and microbial fermentation of yak fed with different dietary energy levels. Moreover, we also checked the effects of different dietary energy levels on VFA transporter genes in the rumen epithelium of yak.

We observed significant increases in acetate, propionate, butyrate, and valerate concentrations as dietary energy levels increased; however, no significant increase in the concentrations of isobutyrate and isovalerate was observed. Among all VFAs, acetate, propionate, and butyrate are particularly known to be associated with animal feed efficiency. Acetate and butyrate are used for fat synthesis and energy supply, while propionate is the main source of glucose for ruminants (Den Besten et al., 2013). We recorded high feed efficiency in yaks from YM and YH groups, which could be explained by a significant increase in concentrations of VFA with the resulting increase of metabolizable energy content of the feed (Yang et al., 2019). High concentrations of acetate, propionate, and valerate in rumen of high milk-producing Holstein dairy cows were reported (Xue et al., 2019). High concentrations of butyrate and a tendency for greater total VFA concentration and acetate have been reported in efficient animals (Guan et al., 2008). A study conducted on beef cattle reported the association of concentrations of rumen VFA and feed efficiency (Li et al., 2019). Increase in concentrations of rumen VFA is known to be associated with lower rumen pH and decrease in animal performance (Koike and Kobayashi, 2001; Klieve et al., 2003). We recorded a decline in rumen pH with an increase in VFA concentrations, but no significant difference in feed efficiency was recorded between YM and YH groups. One reason could be that rumen pH in this study did not cross the threshold value for rumen acidosis that is associated with low feed efficiency in ruminants (Krause and Oetzel, 2006). To maintain rumen pH, VFAs are transported from the rumen to the blood through VFA transporters. The increase in VFA concentrations in yak rumen presumably enhanced the mRNA expression of transporter genes that are involved in VFA absorption and transportation, that is, *MCT1*, *AE2*, *DRA*, and *PAT1*, in the rumen epithelium. The increases in transcripts abundances of *MCT1*, *AE2*, *DRA*, and *PAT1* were also reported in goats fed a high-energy diet (Yan et al., 2014). The concentrations of different VFAs in the rumen associate with the composition of microbial community (Griswold et al., 1999). We then evaluated the differences in rumen bacterial community of yak and their association in improving feed efficiency. In this study, alpha diversity indices did not differ significantly between treatments. Studies have shown host specificity of rumen bacterial community, which might have played an important role in this study (Wiener et al., 2006; Clemmons et al., 2019; Li et al., 2019). However, we recorded significant differences in beta diversity supporting the variations between the microbial communities of each group. Of the 26 bacterial phyla identified in yak rumens, Firmicutes and Bacteroidetes showed high relative abundances. The presence of these phyla has also been reported in the rumen of different ruminants, indicating their importance in the rumen (Ley et al., 2008). These two phyla are considered as an important microbial parameter to assess the energy requirements of ruminants (Xue et al., 2017). Firmicutes are known to be involved in cellulose, hemicellulose, starch, and oligosaccharide degradation as well as in acetate, propionate, and butyrate production. Moreover, Bacteroidetes are known for their role in oligosaccharide hydrolysis and acetate and propionate production. Yaks fed with different feed types showed high relative abundances of Bacteroidetes and Firmicutes in forage and concentrate groups, respectively (Liu et al., 2019). Grazing yaks with low feed efficiency displayed high Bacteroidetes and less Firmicutes abundances in rumen (Zou et al., 2019). The high relative abundance of Firmicutes, increasing the Firmicutes-to-Bacteroidetes ratio, was reported to be associated with efficient feed utilization in animals (Ley et al., 2005; Turnbaugh et al., 2006; Myer et al., 2015). In this study, the high abundance of Firmicutes in the rumen suggests that this shift might have played an important role in affecting feed efficiency. The lack of significant differences in the major phyla between groups might indicate the importance of variations in microbial communities at the genus level. Many of the changes were identified within the phylum Firmicutes at the genus level, which included *Christensenellaceae_R-7_group*, *Ruminococcaceae_NK4A214_group*, *Ruminococcaceae*, *Ruminococcus*, *Lachnoclostridium*, *Saccharofermentans*, *Erysipelotrichaceae*, *Succiniclasticum*, and *Papillibacter*. We recorded significant differences in the relative abundances of phyla Lentisphaerae and Tenericutes with the increase in dietary energy levels. The phylum Lentisphaerae is reported to be associated with changes in feed efficiency, and Tenericutes is essential for rumen homeostasis and health of the host (Mao et al., 2013; Guan et al., 2017). However, little data have been presented on the role of these phyla in improving feed efficiency in the rumen, and the significance of their role in the rumen remains to be determined.

Heatmap analysis revealed significant differences in the relative abundance of specific genera between different dietary energy groups. The relative abundance of genus *Bacteroides* was higher in the YL group. The relative abundance of *Bacteroides* in the rumen is associated with dietary crude fiber content, and their main role is to degrade hemicellulose (Thomas et al., 2011). YL group yaks utilized more roughage, so *Bacteroides* were abundant in their rumen and presumably conducted fiber decomposition. A high relative abundance of *Bacteroides* was also reported in the rumens of goats fed with a low-concentrate diet (Hua et al., 2017). The relative abundances of *Rikenellaceae_RC9_gut_group*, *Ruminococcaceae*, *Ruminococcus*, *Lachnoclostridium*, and *Saccharofermentans* were higher in the YM group. *Rikenellaceae_RC9_gut_group* are known to indicate animal health and associated with obesity (Clarke et al., 2013). *Rikenellaceae_RC9_gut_group* are known for their role in producing acetate and propionate as fermentation end products (Holman and Gzyl, 2019). Studies showed the involvement of *Ruminococcaceae* in fiber degradation and biohydrogenation in the rumen (Gagen et al., 2015; Opdahl et al., 2018). Diet supplemented with linseed oil reported the increase in relative abundance of genus *Ruminococcus* in the rumen of Yanbian Yellow Cattle (Li et al., 2015). *Ruminococcus* is also known as an important genus involved in acetate production (Jeyanathan et al., 2014). In this study, *Ruminococcus* showed a negative correlation with acetate, although a high relative abundance of *Ruminococcus* was identified in the YM group. The increase in the relative abundance of *Ruminococcus* might be stimulated by resistant starch and crude fat present in the diet due to its characteristic amylolytic and lipolytic activities (Krause et al., 2003; Ferrario et al., 2017). In the YH group, *Erysipelotrichaceae*, *U29-B03*, *Succiniclasticum*, *Prevotella*, *Treponema*, *Victivallis*, and *Papillibacter* were the most dominant genera. *Treponema* is mainly involved in the degradation process of soluble carbohydrates (Stanton and Canale-Parola, 1980), and *Succiniclasticum* is a starch-degrading bacterium that degrades dietary starch, produces acetic acid, and succinic acid, and converts succinic acid to propionic acid (Fernando et al., 2010; Huws et al., 2015). In beef cow rumens, *Treponema* and *Ruminobacter* showed high abundances when a high-grain diet was provided, which suggested that these bacteria were present in response to adaptation to the high-grain diet (Chen et al., 2011). *Prevotella*, another important genus in the rumen, utilizes hemicellulose and plays important roles in protein metabolism and starch degradation (Griswold et al., 1999; Ramšak et al., 2000). The replacement of *Ruminococcus* and *Butyrivibrio* by *Prevotella* has been broadly reported during adaptations of rumen microbiota to high-energy diets (Callaway et al., 2010; Fernando et al., 2010; Pitta et al., 2014; Derakhshani et al., 2017). The high relative abundance of *Prevotella* was also reported in a high-energy-diet group compared to groups fed medium- and low-energy diets in white Cashmere goats (Xufeng, 2015). In cows, *Prevotella* abundance was significantly increased when the diet was switched from low grain to high grain (Fernando et al., 2010). An *in vitro* study conducted with sheep rumen fluid reported the increase in *Prevotella* with supplementation with plant and marine oils (Vargas et al., 2017). In this study, YH yaks had the highest rumen starch contents; furthermore, the high abundance of *Prevotella* and rapid starch degradation in the rumen, which increased acetic acid and propionic acid yields, indicated a positive correlation of *Prevotella* with VFA production. Dietary energy levels clearly influenced the rumen bacterial community and increased the relative abundances of non-structural carbohydrate-degrading bacteria. Here, we studied rumen bacterial community by using rumen fluid which lacks undigested solid fiber particles. Whole rumen content including liquid and solid fractions should be studied to better explore the bacterial composition.

Correlation analysis indicated a cluster of bacteria positively correlated with VFA and transcript abundance of VFA transporters mainly belonging to Firmicutes and Bacteroidetes, including *Erysipelotrichaceae UCG-004*, *Lachnospira*, *Prevotellaceae UCG-003*, *SP3-e08*, *Prevotella*, *U29-B03*, and *Christensenellaceae_R-7*, signifying their importance in VFA synthesis and energy generation. *Christensenellaceae_R-7* and *Rikenellaceae_RC9*, the dominant genera in this study, displayed positive and negative correlations with all ruminal VFAs, respectively. We also identified that *E. ruminantium* and *Victivallis* displayed positive correlations with VFAs and negative correlation with VFA transporter genes. The correlation analysis in this study was based on a combined dataset; the lack of significant correlations between some bacterial taxa with VFA and their transporter genes does not suggest that those bacterial taxa are unimportant. More work is needed to explore the correlations, because a small number of species might have a strong impact on rumen fermentation parameters (Hanage, 2014; Patra and Yu, 2014; Shabat et al., 2016). It must be taken into account that although the variations in genera and their putative functions in the rumen could be correlated with the observed differences in the phenotypes, their contribution in feed efficiency is still not clear and require more study.

## Conclusion

In conclusion, the results presented here provide new information regarding the effects of different dietary energy levels on feed efficiency, rumen fermentation, transcript abundance of VFA transporters, and microbial communities. The increase in dietary energy levels enhanced the feed efficiency of yaks. Increase in the concentration of VFAs and transcript abundance of VFA transporters was also recorded with an increase in dietary energy levels. The dietary energy levels affected the microbial diversity, and the increase in energy levels increased the relative abundance of Lentisphaerae and Tenericutes at the phylum level and increased the relative abundance of *Erysipelotrichaceae*, *U29-B03*, *Succiniclasticum*, *Prevotella*, *Treponema*, *Victivallis*, and *Papillibacter* at the genus level. These findings are of great importance for the targeted improvement of nutrient levels in ruminants.

## Data Availability Statement

The datasets generated for this study can be found in the http://www.ebi.ac.uk/ena/data/view/PRJEB34298.

## Ethics Statement

The animal study was reviewed and approved by guidelines for the Care and Use of Laboratory Animals, Lanzhou Institute of Husbandry and Pharmaceutical Sciences, Chinese Academy of Agricultural Sciences, China. Written informed consent was obtained from the owners for the participation of their animals in this study.

## Author Contributions

XD, PY, and RL designed the research. AA, CY, QK, JZ, CL, ZL, and MD conducted the experiment and collected the data. AA and CY analyzed the data. AA wrote the first draft of manuscript text. XD, CY, QK, JZ, and JH revised the manuscript. All authors read and approved the final manuscript.

## Conflict of Interest

The authors declare that the research was conducted in the absence of any commercial or financial relationships that could be construed as a potential conflict of interest.

## Footnotes

1 https://www.drive5.com/usearch/manual/uclust_algo.html

2 http://www.ebi.ac.uk/ena/data/view/PRJEB34298
