# Supplementary material for: Effects of Dietary Energy Levels on Rumen Fermentation, Microbial Diversity, and Feed Efficiency of Yaks (Bos grunniens)
Source: Front Microbiol. 2020 May 15;11:625. doi: 10.3389/fmicb.2020.00625 (PMC7326093; doi:10.3389/fmicb.2020.00625)
Supplement: Supplementary file 1 [file Data_Sheet_1.docx]

Supplementary Materials

Table S1. Primer sequences with accession number and product size of the genes associated with VFA transportation

| Genes | Primer sequence (5’→3’) | | Accession | Product size |
| --- | --- | --- | --- | --- |
| MCT1 | F | CTGGCAGCACCTTTATC | HM061149.1 | 109bp |
|  | R | GGTCCATCAGCGTTTC |  |  |
| AE2 | F | CCAAGTCGGCTCAGGAT | NM_001205664.1 | 148bp |
|  | R | CGGAAGTCGCTTAGGTAGTG |  |  |
| DRA | F | TTGTGCCGCTGTTCT | BC134586.1 | 103bp |
|  | R | CACTGAGGAGCCATTC |  |  |
| PAT1 | F | CCAGTGCTTCCCTGTGAG | BC123616.1 | 151bp |
|  | R | CGCCTTGGGTAGGTCTTG |  |  |
| β-actin | F | ACCATCGGCAATGAGCG | XM_005887322.2 | 150bp |
|  | R | CACCGTGTTGGCGTAGAG |  |  |


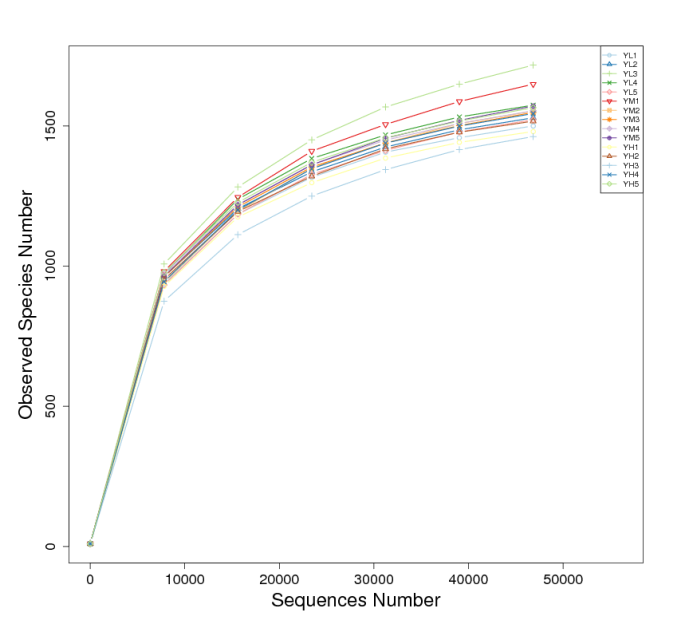


Figure S1. Rarefaction curve of observed species number at 97% similarity level of index of different samples


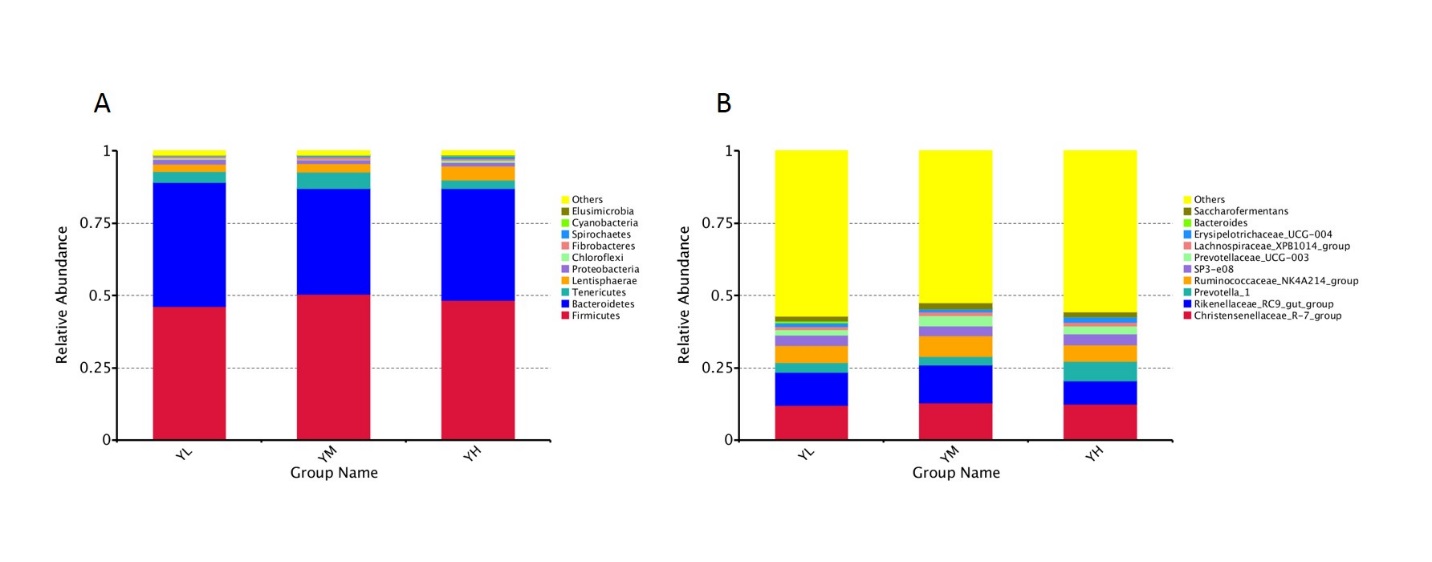
 Figure S2. Influence of different dietary energy treatments on relative abundance of bacteria in yak rumen at (A) Phylum and (B) Genus levels. Y-axis shows the relative abundance of bacteria, while Y-axis displayed three different dietary groups.
